# A 20-SNP Panel as a Tool for Genetic Authentication and Traceability of Pig Breeds

**DOI:** 10.3390/ani12111335

**Published:** 2022-05-24

**Authors:** Riccardo Moretti, Andrea Criscione, Federica Turri, Salvatore Bordonaro, Donata Marletta, Bianca Castiglioni, Stefania Chessa

**Affiliations:** 1 Department of Veterinary Science, University of Turin, 10095 Turin, Italy; riccardo.moretti@unito.it; 2 Department of Agriculture, Food and Environment, University of Catania, 95131 Catania, Italy; a.criscione@unict.it (A.C.); s.bordonaro@unict.it (S.B.); donata.marletta@unict.it (D.M.); 3 Institute of Agricultural Biology and Biotechnology, National Research Council, 26900 Lodi, Italy; turri@ibba.cnr.it (F.T.); castiglioni@ibba.cnr.it (B.C.)

**Keywords:** pig breeds, traceability, molecular markers, SNP

## Abstract

**Simple Summary:**

Given the high economic and qualitative values of local-breed meat products, it is not uncommon that substitution or mislabeling (either fraudulent or accidental) occurs at the market level. Therefore, to protect the interests of both producers and consumers, a reliable traceability tool should be developed. Nowadays, traceability usually relies on physical labeling systems (e.g., ear tags, tattoos, or electronic transponders). These systems do not, however, have good performances when dealing with carcasses or processed meat products. Molecular markers (i.e., based on the DNA sequence) can be a solution, since DNA is easily extracted from a wide variety of animal products and parts, and is not degraded during processing, even at the high temperatures involved. The aim of this study was to identify a small number of DNA mutations for breed-traceability purposes, in particular of the Italian Nero Siciliano pig and its derived products. A small panel of 12 DNA mutations was enough to discriminate Nero Siciliano pigs from other pig breeds and from wild boars.

**Abstract:**

Food authentication in local breeds has important implications from both an economic and a qualitative point of view. Meat products from autochthonous breeds are of premium value, but can easily incur fraudulent or accidental substitution or mislabeling. The aim of this study was to identify a small number of SNPs using the Illumina PorcineSNP60 BeadChip for breed traceability, in particular of the Italian Nero Siciliano pig and its derived products. A panel of 12 SNPs was sufficient to discriminate Nero Siciliano pig from cosmopolitan breeds and wild boars. After adding 8 SNPs, the final panel of 20 SNPs allowed us to discriminate all the breeds involved in the study, to correctly assign each individual to its breed, and, moreover, to discriminate Nero Siciliano from first-generation hybrids. Almost all livestock breeds are being genotyped with medium- or high-density SNP panels, providing a large amount of information for many applications. Here, we proposed a method to select a reduced SNP panel to be used for the traceability of pig breeds.

## 1. Introduction

In recent decades, consumers and producers have paid increasing attention to food quality and safety, and to traditional foods. Food authenticity has become one of the most important concerns through the entire production chain. To date, traceability has relied on a labeling system that should ensure a connection between the final processed product and the animal source itself [1]. This system is traditionally based on the use of labels such as ear tags, tattoos, or electronic transponders [2], making it difficult to keep track of, for example, every piece of a carcass. DNA analysis represents a solution to overcome this difficulty, since DNA can be easily extracted from a wide variety of animal products and parts [3]. Recently, microsatellites [4,5,6], SNPs [7], mitochondrial DNA [8], and QTLs [9] were used for the identification of different species or breeds. SNPs are the most favored marker due to their widespread presence in the whole genome and the high reliability and automation of SNP genotyping assays [10]. Different studies have investigated the possibility of using SNP panels for traceability purposes: a 90-SNP panel was used to evaluate geographical and individual traceability of 24 European cattle breeds (with an average assignment probability higher than 90%) [11]; and a 6-SNP panel in candidate genes for meat and growth traits was proposed to differentiate eight Chinese and Argentine breeds, allowing the discrimination of their geographical origin with a correct assignment of more than the 70% of individuals [12]. Regarding pig breeds, while searching for breed-specific SNPs using the Illumina PorcineSNP60 BeadChip, Ramos et al., (2011) [7] identified a total of 193 private SNPs in Duroc (DU), Landrace (LR), Large White (LW), Pietrain (PI), and Wild Boar (WB). These SNPs were used to assign individuals to their correct breed, obtaining a 99% assignment rate. However, this panel cannot be applied in other breeds, and has a genotyping cost not yet affordable for routine analyses, especially for local breeds.

The interest in local breeds is growing worldwide, as demonstrated by the large number of studies published in recent years [13,14,15]. This is due to their rusticity, their adaptability to the environment, the peculiar characteristics of the derived products and their connection to tradition, and the area of production. Many Protected Designation of Origin labels were established in the European Union involving monobreed products, with processing and quality thresholds that must be respected during all of the production steps (Regulation (EU) No. 1151/2012). Since such products usually gain a premium price, fraud threat for the products derived from local breeds is a pressing topic in Italian and European markets, thus making an efficient and reliable traceability system a priority [14].

Nero Siciliano (NS) is an Italian autochthonous breed, reared in the northeastern part of the island of Sicily. NS pigs are bred on small farms, mainly under extensive management systems [16], and therefore accidental crossbreeding with WB is a widespread problem. Hybrids are fertile and often more aggressive and voracious than purebred pigs. After a decline during the 20th century, due to the wide spread of cosmopolitan breeds (e.g., LR and LW), which have a higher meat yield and growth rate, NS rearing has seen an important increase in the last 10 years thanks also to the creation of a Protected Designation of Origin label for its meat and other related products [17]. An interest in conserving this breed was also demonstrated by the creation of a semen cryobank as a genetic reserve of the NS pig breed, which now consists of 1455 semen straws [18].

The aim of this work was to identify a small panel of SNPs usable for distinguishing NS from the main cosmopolitan breeds reared in Italy (LW, LR, and DU) and WB, as a tool for the traceability of its meat and related products. We also simulated first-generation (F1) hybrids to check whether a small SNP panel was able to discriminate purebred animals from hybrids, as a first step in the development of a system to identify frauds intended as the use of crossbreds or meat mixtures marketed as monobreed products.

## 2. Materials and Methods

### 2.1. Breeds, Sampling, and Genotyping

Nero Siciliano (NS) is an ancient Italian autochthonous black pig breed (the presence of which is described by ancient documents and dated by fossils at least to the 7–6th century BC), reared in the northeastern part of Sicily on small traditional farms, mainly under extensive management systems. NS is characterized by slow growth, a high meat quality, and adaptation to harsh conditions [19]. It has a black coat, and its meat is used to produce high quality products, including salami and cured ham. After a decline in the 20th century, a strategy for the valorization of its products and the establishment of the Italian herdbook in 2001 helped in the renewal of the breed.

A total of 93 blood samples were collected from NS pigs reared on 22 farms well distributed in the natural park of the Nebrodi Mountains, in the northeastern part of Sicily. Blood-sample collection was performed via jugular vein puncture into EDTA vacutainer tubes with a 21G needle, contextually to the routine sanitary screening of the National Animal Health Services and in accordance with the general recommendations of respect for animal welfare; all efforts were made to minimize animal suffering. Sampling was performed to avoid strongly related animals, based on information obtained from both breeders and genealogy data (when available).

Genomic DNA was extracted using a commercial kit (NucleoSpin Blood, Macherey-Nagel, Düren, Germany) following the manufacturer’s protocol. After qualitative and quantitative checks (NanoDrop 1000 Spectrophotometer, Thermo Fisher Scientific Inc., Waltham, MA, USA), the extracted DNA was genotyped in outsourcing using Neogen Corporation’s GeneSeek (Lincoln, NE, USA) with the PorcineSNP60 BeadChip v2 (Illumina, San Diego, CA, USA).

Genotyping data of other breeds were obtained from previous studies to obtain a more representative sample of these breeds: 65 Italian samples from 3 cosmopolitan breeds analyzed by Chessa et al. (2011) [20] and consisting of 24 LW, 17 LR, and 24 DU; and 88 Northwest European WB samples and 60 samples from 3 cosmopolitan breeds (20 LW, 20 LR and 20 DU) tested by Goedbloed et al., (2013) [21]. Thus, the final data set consisted of a total of 306 individuals belonging to four domestic breeds and wild boar. Individuals from previous studies were genotyped with the Illumina PorcineSNP60 BeadChip v1.

F1 hybrids were simulated using HYBRIDLAB v1.1 [22], which created multilocus F1 hybrid genotypes between two populations. Hybrids were therefore generated as F1 individuals of the following crossings: NS × DU, NS × LR, NS × LW, NS × WB, WB × DU, WB × LR, WB × LW, DU × LR, DU × LW, and LR × LW; 20 and 10 individuals per hybrid type were generated by the software and used in the HTP and HVP, respectively.

### 2.2. Data Analysis and SNP Selection

The NS 60K SNP panel data was edited using PLINK software [23] to remove individuals with a call rate <95% and SNPs with a call rate <95% and deviance from the Hardy–Weinberg equilibrium (*p* < 0.01 Bonferroni-corrected, Wiggans et al., 2009 [24]). Subsequently, for the different datasets, SNPs not in common with the 45,720 SNPs genotyped by Goedbloed et al., (2013) [21] were also removed together with a total of 986 SNPs present in the PorcineSNP60 BeadChip v2 and not in the previous v1. Rare alleles were not removed from the dataset, since they could be useful for traceability purposes.

Where not specified, data analyses were conducted in the R base environment [25]. The relationships between individuals and breeds were checked via multidimensional scaling (MDS) using the GenABEL 1.8-0 R package [26] to build a matrix of average identity by state (IBS) of the analyzed individuals and visualize the distances with the relative two-dimensional plot.

Different statistical selection methods (e.g., Wright’s Fst [27] and principal component analysis [28]) are available to determine which genetic markers are the most informative in order to discriminate among different populations. Widely applied both in human [29] and animal [30] genetics, one of the most used statistical selection methods is the delta statistic (i.e., a higher distance between the same allele frequencies in two different breeds). Allele frequencies for each SNP per breed were therefore calculated using PLINK software and compared with pairs of breeds to determine the smallest SNP panel with the greatest discriminant power using the delta statistic, in order to provide a cheap and effective diagnostic tool available for routine analysis. The delta statistic is one of the most used measures of marker informativeness, and is widely used in human genetics: its value is given by |PA_i_−PA_j_|, where PA_i_ and PA_j_ are the frequencies of allele A in two different populations (i.e., i and j). Pairwise comparisons were performed and subsequently analyzed.

After selecting the most informative SNP, data were further filtered using PLINK software to remove individuals with missing genotypes. To verify the ability of the selected SNPs to discriminate among breeds and F1 hybrids, canonical discriminant analysis (CDA) was used as already described by Dimauro et al., (2013) [31]. Such analysis is able to maximize differences among predefined groups by extracting a set of linear combinations of the original variables and then, by means of discriminant analysis, elaborating a discriminant function able to assign new observations to groups. We applied the same procedure using the SNPs selected with the delta statistic as original variables. Training/validation tests were performed using the R packages candisc 0.6-5 [32] and MASS 7.3-35 [33]. We thus set 4 panels of individuals (Supplementary Table S1) composed of purebred and of purebred and hybrid subjects: a first training population consisting of 275 purebred individuals (purebred training population, PTP) remaining after PLINK’s pruning of missing genotypes in the original population. A second training population (hybrid training population, HTP) included, in addition to the PTP, 200 hybrid individuals. The corresponding validation populations were composed of 200 purebred individuals obtained by generating 40 purebreds for each breed (purebred validation population, PVP), and of 100 hybrid individuals in addition to the PVP obtained by simulating 10 hybrids for each F1 crossing (hybrid validation population, HVP), respectively.

## 3. Results

### 3.1. Genotyping

The mean genotyping rate for the NS pigs was 98.5%. In particular, 90 subjects out of 93 had a call rate (CR) higher than 99%, and 74 were higher than 99.5%. Only one individual had a CR of 62% and was therefore removed. After data cleaning of the NS dataset, 58,232 of the 61,565 SNPs included in the PorcineSNP60 BeadChip v2 were retained. After merging this dataset with the one including the LW, LR, DU, and WB populations, and removing SNPs that were absent in v1 of the chip, 43,697 SNPs were available for the subsequent analyses.

### 3.2. Multidimensional Scaling

**Figure 1:**
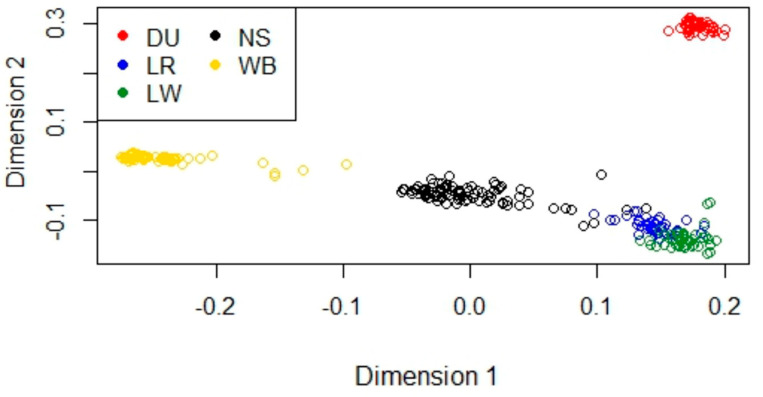
Multidimensional Scaling. Analysis of Nero Siciliano (NS), Landrace (LR), Large White (LW), Duroc (DU), and Wild Boar (WB).

Before proceeding with SNP selection for discriminant analyses, a multidimensional-scaling approach was applied to the cleaned dataset (43,697 SNPs and 306 pigs: 93 NS, 44 LW, 37 LR, 44 DU, and 88 WB) in order to assess group clusterization of the individuals: close or overlapping clusters involving a genetic similarity amid populations. As shown in Figure 1, the first dimension separated the clusters by the wild-vs.-domestic pigs, with NS clustering halfway between the WB and domestic pig breeds, due to both a recent domestication event and lesser selection pressure applied to this breed. The second dimension differentiated the breeds by coat color, centering the wild-type black coat and discerning from the reddish coat of the DU pigs to the white of the LR and LW cluster. From a two-dimensional point of view, the WB and DU pigs instead clustered separately from each other, while the LR and LW pigs clustered very closely between them and not far from the NS pigs. The proximity of these cosmopolitan breeds with NS could derive from an ascertainment bias due to the breeds used to design the chip itself: SNPs chosen to be represented on the chip derive from the five worldwide diffused commercial breeds (LR, LW, DU, PI and WB) [34]. No NS or other minor breeds’ individuals were present in the dataset used for SNP selection, leading to a possible smaller capability of the assay in discerning these breeds. In addition, the higher genetic similarity of the LR and LW breeds could derive from the similar strong directional selection they experienced, thus leading to the close clusterization of the two white breeds.

**Figure 2:**
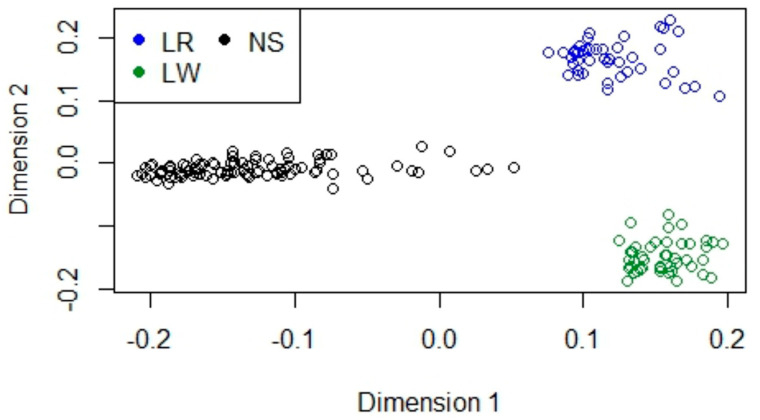
Multidimensional Scaling. Analysis of Nero Siciliano (NS), Landrace (LR), and Large White (LW).

To deeply investigate this feature, we performed an MDS analysis using only the LR, LW and NS breeds (Figure 2): the NS cluster was well defined for both dimensions 1 and 2, while LR and LW pigs were very well discernible only for dimension 2. Nevertheless, the three breeds were well distinguishable when compared without other breeds.

### 3.3. SNP Selection

We ranked the SNP markers based on their genetic information content, and therefore they were ordered by decreasing level of delta to identify the more informative ones, as suggested in Wilkinson et al., (2011) [14]. According to the delta-based approach, we selected a 12-SNP panel including the 3 SNPs with a higher delta per comparison between NS-LR and NS-LW, the 2 SNPs with a higher delta for NS-WB comparison, and 1 SNP for NS-DU comparison (Supplementary Table S2). The remaining three SNPs of the panel were selected according to the results of MDS analysis in order to increase the discernibility of the near-clustering breeds (e.g., a better discerning of LR from LW). Fewer SNPs for NS-WB and NS-DU comparison were needed for their clear discrimination due to the above-mentioned MDS-assessed genetic differences. A second subset of 20 SNPs (Table 1) was selected and added to the previous panel of 8 SNPs with a higher delta per comparison between the other breeds compared by couples in order to improve the breed assignment when including hybrids. In particular, the eight added SNPs were chosen to help distinguish the more biased breeds (e.g., LR, LW, and their hybrid crossing).

### 3.4. Canonical Discriminant Analysis

To perform the CDA, the 306-sample dataset was pruned of the individuals with a missing genotype. A total of 275 animals had no missing genotypes when considering the 20-SNP panel, and were therefore used as the training population as well for the 12-SNP panel to obtain a better comparison of the performances of the two SNP panels.

The 12-SNP panel was first tested to assess if it was sufficient to discriminate correctly among the analyzed breeds; 98.2% of the individuals in the training population (PTP dataset, composed of the initial 275 individuals fully genotyped for the 12 selected SNPs) were correctly assigned. The incorrectly allocated individuals were one DU (assigned to WB), two LR (one assigned to LW and one to WB) and two LW (assigned to LR). All of the NS pigs were attributed to the correct cluster, with an average probability of assignation of 98.1%. Detailed results of the performances of the 12- and 20-SNP panels in the linear CDA can be found in Table 2.

A total of 97% of the 200 individuals in the relative PVP were also correctly assigned, along with a 100% total for the correct assignment of NS pigs. The incorrectly allocated individuals were two LR (one assigned to LW and one to WB again) and four LW (all confused with LR). The average probability of assignation of individuals to the right cluster was 97.1% for the entire PVP and 99.8% for NS pigs (unpublished data).

Using the same 12-SNP panel, a discriminant analysis was performed on the HTP dataset (275 real pigs plus 200 simulated hybrids labeled as HY): 85.3% of the individuals in the training population were correctly assigned to their own cluster; in particular, 89 NS pigs out of 92 were correctly assigned, while 3 individuals were allotted to the HY cluster. The validation phase we ran on the HVP dataset resulted in an average correct assignment for 87% of the individuals, with 100% correct assignation for the simulated NS to NS cluster. The average probability of assignation per individual to the right cluster was 83.1%: 91.6% for purebred individuals, 97.1% for purebred NS, and 66.1% for hybrid individuals. Thus, hybrid identification was a more complicated task.

To increase the performance when including hybrids, 8 SNPs were added, and the obtained 20-SNP panel was then analyzed. The first analysis was performed using the PTP dataset: the linear discriminant analysis and its subsequent prediction applied to the dataset resulted in a perfect attribution (100%) of all the individuals to their respective breeds. Validation-phase testing was then performed using the PVP dataset, including the simulated purebred individuals, and also resulted in a perfect assignment (100%) for all the individuals. The second analysis, conducted using the HTP dataset for the training population resulted in a correct attribution for 92.2% of the individuals; in particular, 87 out of 92 NS pigs were correctly identified as purebred, while the remaining 5 were assigned the hybrid cluster. The following validation phase correctly identified 94.7% of the individuals. All the purebred individuals in the validation dataset were correctly attributed to their breed’s cluster, while only 84 out of 100 (84%) hybrids were correctly assigned, with the others being allotted to one of their parental breeds (Supplementary Table S3). For the validation population, the average probability of assignation was 90.8%, with 97.9% for purebred individuals (98.1% for NS pigs) and 76.7% for hybrid individuals, with an average probability assignment of 65% and 85% for the incorrectly and correctly assigned individuals, respectively. Considering such assignation probability, we fixed a threshold of 65% as the minimum assignation probability of assigned individuals to a breed in a blind test: individuals with a higher probability than this threshold were assigned to the cluster identified by the CDA, while individuals with a lower one were not assigned to any categories and assumed to be hybrids, so they were reassigned as hybrids. Using this threshold, only two purebred individuals (one LR and one LW), and five hybrids (three LR × LW and two LR × WB) failed in the assignment to the correct group. The six NS hybrids that were assigned as purebreds by the canonical discriminant analysis had a mean assignation probability of 54.93%, which meant that they could be attributed as a purebred only for about a half of their genotype, and should probably be considered hybrids. Thus, the use of the threshold helped us to better identify the hybrids, and with it, we reached a correct attribution rate of 97.7% (2.3% error rate). As expected from the MDS approach, most of the errors involved the LR and LW breeds, whereas neither NS nor their hybrids were incorrectly assigned (Table 3).

## 4. Discussion

When dealing with traceability, the most interesting SNP to be used should be a private allele. Finding a private allele in local breeds is time-consuming and costly, and potentially requires whole-genome sequencing of enough representative individuals of each involved breed. If some potential private alleles are discovered, then they should be tested in a larger population to verify that they are actually present only in one breed. Thus, using commercial medium- or high-density SNP panels is a cheaper and simpler approach that could be used on potentially all breeds of a species. One of the most difficult and controversial aspects in selecting a reduced number of SNPs when starting with medium- or high-density SNP panels consists of choosing the most suitable way of selecting the SNPs. Many different statistical selection methods have been described [27,28], but we decided to use the delta statistic (the higher distance between the same allele frequencies in two different breeds), since the main purpose was to magnify the differences between the NS and the other analyzed populations. Moreover, if the chosen SNPs are not subjected to selection, the allelic frequencies should remain almost unchanged in the breeds in the following generations, guaranteeing that the selected reduced SNP panel will serve as a stable traceability instrument.

Using the 12-SNP panel, we were able to correctly assign 98.2% of the individuals in the training population and 97% of the validation population. In particular, the Nero Siciliano pig was discerned from both cosmopolitan breeds and wild boars, with a 100% attribution of individuals to the correct cluster. The incorrectly allocated individuals belonged to the cosmopolitan breeds LW, LR, and DU, and none of them were assigned to NS, thus confirming that our method of selecting the SNPs and the selected SNPs were appropriate as a traceability instrument for the NS breed. Using the 20-SNP panel, we were able to correctly assign 100% of the individuals to their breeds.

Hybrid identification was obviously a more complicated task. Using the 20-SNP panel, we were able to correctly assign 92.2% of the individuals in the training population and 94.7% of the validation population, with all the purebred individuals correctly assigned in the second dataset. Moreover, when adding an assignation probability threshold of 65% to confirm the purebreds, the correctly assigned individuals totaled 97.7%, and all the Nero Siciliano hybrids were assigned to the correct cluster. This is of great value in potentially preventing frauds intended as meat mixtures that are passed off as monobreed products. Finally, when considering the hybrids and using the assignation probability threshold of 65% to reduce the assignation errors, the individuals that were still incorrectly assigned were related to cosmopolitan breeds. The higher overestimation of the expected heterozygosity for populations involved in the discovery process than for the population not included in the SNP selection for the original array [35] actually could make it more difficult to find a few SNPs that help in distinguishing better cosmopolitan breeds

## 5. Conclusions

The genetic authentication of animal products from local breeds is a challenging task for geneticists, but the development of an easy-to-use tool for breed traceability would protect consumers from frauds and preserve animal genetic resources, providing a higher gain to the breeders. NS, the pig breed analyzed in this study, is an endangered population to be safeguarded in its breeding area, and the authentication of its derived meat products will help to maintain the local sustainable rearing system and add value to its monobreed products. Previous studies used panels with a low number of SNPs to compare different breeds, showing that it was possible to use them to differentiate many breeds [36]. We further reduced the number of SNPs to be analyzed to 20, and also applied this number to discriminate purebreds from hybrids. This panel could easily be tested in many laboratories using one of the many available low-cost genotyping techniques (KASP^TM^, MALDI-TOF-MS, target SNP-seq, etc.).

With our approach of selecting the SNPs while considering the delta statistic, the ascertainment bias of an array design that usually negatively affects population genetic estimators when dealing with breeds not involved in the SNP selection seemed to be bypassed, allowing the correct assignation of these breeds. The SNP selection approach used in this study is therefore not limited to NS, and also could be applied to other autochthonous pig breeds to select the most informative SNPs from a commercial genotyping chip. Furthermore, this approach also could be applied to local breeds of species other than porcine ones that face the same threats.

## Figures and Tables

**Table 1:** Panel of 20 SNPs selected using statistical delta. Position refers to Sscrofa 10.2 assembly. The 12 SNPs used to discriminate Nero Siciliano breed from commercial breeds and Wild Boar are bolded.

SNP	SNP ID	Chromosome	Position	Breed ^1^	Δ with NS
MARC0027620	rs81222690	1	17,004,757	DU	0.621
INRA0002279	rs326314161	1	57,648,581	LW	0.546
**ASGA0004735**	rs80788426	1	159,034,424	LW	0.783
ASGA0007653	rs81352517	1	300,830,758	LW	0.506
**H3GA0006564 ^2^**	rs81357620	2	42,213,270	NS	0.701
**CASI0009067**	rs329578899	2	108,053,445	WB	1.000
ASGA0021239	rs81001550	4	105,898,886	DU	0.939
**ALGA0027544**	rs80958781	4	113,430,367	LW	0.731
M1GA0006536	rs80935048	4	133,828,395	LR	0.536
H3GA0016973	rs81385751	5	85,729,923	LW	0.723
**MARC0038980**	rs81232179	8	51,070,662	LR	0.761
**INRA0029816**	rs327195280	8	51,235,665	LR	0.763
**ALGA0047912**	rs81400622	8	57,588,232	LW	0.743
MARC0042228	rs81234311	8	139,592,943	WB	0.491
**ALGA0065765 ^2^**	rs81433050	12	27,112,308	NS	0.758
**ASGA0069722**	rs81453203	15	63,655,984	DU	0.932
**ALGA0107321**	rs81335037	15	141,212,829	DU	0.779
**ASGA0095426**	rs81314826	15	149,495,100	WB	0.953
INRA0052808	rs342665431	17	17,548,564	LR	0.607
**DBMA0000205**	rs45432506	17	20,690,224	LR	0.788

^**1**^ Breeds: NS = Nero Siciliano; DU = Duroc; LW = Large White; LR = Landrace; WB = Wild Boar. ^**2**^ SNPs characterizing NS from all the other breeds tested.

**Table 2:** Total assignation rate of individuals for 12-SNP and 20-SNP panels.

Breed ^1^	12 SNP				20 SNP			
	PTP ^2^	PVP ^2^	HTP ^2^	HVP ^2^	PTP ^2^	PVP ^2^	HTP ^2^	HVP ^2^
NS	92/92 (100%)	40/40 (100%)	89/92 (96.7%)	40/40 (100%)	92/92 (100%)	40/40 (100%)	87/92 (94.6%)	40/40 (100%)
LW	34/36 (94.4%)	36/40 (90%)	33/36 (91.7%)	33/40 (82.5%)	36/36 (100%)	40/40 (100%)	36/36 (100%)	40/40 (100%)
LR	34/36 (94.4%)	38/40 (95%)	32/36 (88.9%)	38/40 (95%)	36/36 (100%)	40/40 (100%)	34/36 (94.4%)	40/40 (100%)
DU	39/40 (97.5%)	40/40 (100%)	37/40 (92.5%)	36/40 (90%)	40/40 (100%)	40/40 (100%)	39/40 (97.5%)	40/40 (100%)
WB	71/71 (100%)	40/40 (100%)	71/71 (100%)	39/40 (97.5%)	71/71 (100%)	40/40 (100%)	71/71 (100%)	40/40 (100%)
HY	-	-	143/200 (71.5%)	75/100 (75%)	-	-	171/200 (85.5%)	84/100 (84%)
Purebred individuals	*	*	262/275 (95.3%)	186/200 (93%)	*	*	267/275 (97.1%)	200/200 (100%)
Entire population	270/275 (98.2%)	194/200 (97%)	405/475 (85.3%)	261/300 (87%)	275/275 (100%)	200/200 (100%)	438/475 (92.2%)	284/300 (94.7%)

^**1**^ Breeds: NS = Nero Siciliano; DU = Duroc; LW = Large White; LR = Landrace; WB = Wild Boar; HY = F1 hybrids. ^**2**^ PTP = purebred training population; PVP = purebred validation population; HTP = hybrid training population; HVP = hybrid validation population. * Purebred individuals’ assignation values correspond to those of the entire population in the same columns.

**Table 3:** Assignation statistics. The number of individuals correctly assigned by the canonical discriminant analysis (CACDA) and with the addition of the threshold at 65% (CAT65); the wrongly assigned breeds are reported together with the mean and the lowest assignation probability to the right breed.

Breed ^1^	CACDA	Wrongly Assigned Breed	Mean Assignation Probability	Lowest Assignation Probability	CAT65	Wrongly Assigned Breed
NS	40/40		0.981	0.771	40/40	
DU	40/40		0.980	0.757	40/40	
LR	40/40		0.957	0.568	39/40	HY
LW	40/40		0.979	0.618	39/40	HY
WB	40/40		0.999	0.982	40/40	
NS × DU	9/10	DU	0.842	0.390	10/10	
NS × LR	9/10	NS	0.786	0.303	10/10	
NS × LW	10/10		0.775	0.566	10/10	
NS × WB	6/10	2 NS and 2 WB	0.652	0.360	10/10	
DU × LR	10/10		0.948	0.831	10/10	
DU × LW	10/10		0.827	0.534	10/10	
DU × WB	10/10		0.924	0.865	10/10	
LR × LW	4/10	3 LR and 3 LW	0.493	0.054	7/10	1 LR and 2 LW
LR × WB	7/10	1 LR and 2 WB	0.600	0.096	8/10	1 LR and 1 WB
LW × WB	9/10	WB	0.818	0.366	10/10	
Whole population	284/300 (94.7%)				293/300 (97.7%)	

^**1**^ Breeds: NS = Nero Siciliano; DU = Duroc; LW = Large White; LR = Landrace; WB = Wild Boar. The hybrids of each breed’s couple are identified by the two breeds separated by “×”.

## Data Availability

The datasets used and/or analyzed during the current study are available from the corresponding author upon reasonable request.

## Supplementary Materials

The following supporting information can be downloaded at: https://www.mdpi.com/article/10.3390/ani12111335/s1, Table S1: Composition of the populations generated using HYBRIDLAB software; Table S2: Statistical delta per SNP per comparison of breeds; Table S3: Probability of assignation per individual to the different clusters.
