# Supplementary material for: A 20-SNP Panel as a Tool for Genetic Authentication and Traceability of Pig Breeds
Source: Animals (Basel). 2022 May 24;12(11):1335. doi: 10.3390/ani12111335 (PMC9179885; doi:10.3390/ani12111335)
Supplement: Supplementary file 1 [file animals-12-01335-s001.zip › animals-1671755-supplementary.pdf]

Table S1. Composition of the populations generated using HYBRIDLAB software.

**PTP**		**PVP**		**HTP**		**HVP**	
**Breed**	**Num**	**Breed**	**Num**	**Breed**	**Num**	**Breed**	**Num**
DU	40	DU	40	DU	40	DU	40
LR	36	LR	40	LR	36	LR	40
LW	36	LW	40	LW	36	LW	40
NS	92	NS	40	NS	92	NS	40
WB	71	WB	40	WB	71	WB	40
-	-	-	-	Hybrids	200	Hybrids	100
**TOT**	275		200		475		300

PTP = Purebred Training Population; PVP = Purebred Validation Population; HTP = Training Population including F1 hybrids; HVP = Validation Population including F1 hybrids.

Table S2. Statistical delta per SNP per comparison of breeds.

SNP	RS	CHR	NS-LW	NS-LR	NS-DU	NS-WB	LW-LR	LW-DU	LW-WB	LR-DU	LR-WB	DU-WB
MARC0027620	rs81222690	1	0.288	0.416	0.621	0.294	0.704	0.908	0.006	0.205	0.710	0.914
INRA0002279	rs326314161	1	0.546	0.383	0.299	0.201	0.163	0.845	0.748	0.682	0.584	0.098
ASGA0004735	rs80788426	1	0.783	0.096	0.137	0.101	0.687	0.920	0.682	0.234	0.005	0.239
ASGA0007653	rs81352517	1	0.506	0.220	0.288	0.326	0.726	0.794	0.831	0.068	0.106	0.038
H3GA0006564	rs81357620	2	0.701	0.701	0.701	0.701	0.000	0.000	0.000	0.000	0.000	0.000
CASI0009067	rs329578899	2	0.186	0.221	0.239	1.000	0.035	0.053	0.814	0.018	0.779	0.761
ASGA0021239	rs81001550	4	0.026	0.079	0.939	0.037	0.053	0.913	0.063	0.860	0.116	0.977
ALGA0027544	rs80958781	4	0.731	0.049	0.458	0.081	0.682	0.273	0.812	0.409	0.131	0.540
M1GA0006536	rs80935048	4	0.267	0.536	0.283	0.226	0.268	0.550	0.493	0.818	0.761	0.057
H3GA0016973	rs81385751	5	0.723	0.073	0.564	0.220	0.795	0.159	0.943	0.636	0.148	0.784
MARC0038980	rs81232179	8	0.670	0.761	0.397	0.054	0.091	0.273	0.616	0.364	0.707	0.343
INRA0029816	rs327195280	8	0.656	0.763	0.400	0.007	0.107	0.256	0.663	0.364	0.771	0.407
ALGA0047912	rs81400622	8	0.743	0.667	0.049	0.151	0.076	0.792	0.894	0.716	0.818	0.102
MARC0042228	rs81234311	8	0.327	0.389	0.429	0.491	0.716	0.102	0.818	0.818	0.102	0.920
ALGA0065765	rs81433050	12	0.758	0.758	0.758	0.738	0.000	0.000	0.020	0.000	0.020	0.020
ASGA0069722	rs81453203	15	0.000	0.000	0.932	0.000	0.000	0.932	0.000	0.932	0.000	0.932
ALGA0107321	rs81335037	15	0.624	0.518	0.779	0.636	0.106	0.155	0.012	0.261	0.118	0.143
ASGA0095426	rs81314826	15	0.341	0.170	0.023	0.953	0.170	0.318	0.613	0.148	0.783	0.931
INRA0052808	rs342665431	17	0.187	0.607	0.290	0.279	0.420	0.477	0.466	0.898	0.886	0.011
DBMA0000205	rs45432506	17	0.319	0.788	0.026	0.059	0.468	0.293	0.378	0.761	0.846	0.085

Table S3. Probability of assignation per individual to the different clusters in HV validation test with the 20 SNPs panel. The assignation obtained with the canonical discriminant analysis (CACDA) and with the addition of the threshold at 65% (CAT65) are also reported and the animal wrongly assigned with the two methods are bolded.

Breed[1]	Individual	NS	DU	LR	LW	WB	HY	CACDA	CAT65
NS	NS01	0.974	0	0	0	0	0.026	NS	NS
NS	NS02	0.996	0	0	0	0	0.004	NS	NS
NS	NS03	0.999	0	0	0	0	0.001	NS	NS
NS	NS04	0.98	0	0	0	0	0.02	NS	NS
NS	NS05	0.997	0	0	0	0	0.003	NS	NS
NS	NS06	0.956	0	0	0	0	0.044	NS	NS
NS	NS07	0.963	0	0	0.007	0	0.029	NS	NS
NS	NS08	1	0	0	0	0	0	NS	NS
NS	NS09	1	0	0	0	0	0	NS	NS
NS	NS10	0.998	0	0	0	0	0.002	NS	NS
NS	NS11	1	0	0	0	0	0	NS	NS
NS	NS12	0.995	0	0	0	0	0.005	NS	NS
NS	NS13	0.994	0	0	0	0	0.006	NS	NS
NS	NS14	0.998	0	0	0	0	0.002	NS	NS
NS	NS15	0.991	0	0	0	0	0.009	NS	NS
NS	NS16	0.993	0	0	0	0	0.007	NS	NS
NS	NS17	1	0	0	0	0	0	NS	NS
NS	NS18	0.994	0	0	0	0	0.006	NS	NS
NS	NS19	1	0	0	0	0	0	NS	NS
NS	NS20	0.999	0	0	0	0	0.001	NS	NS
NS	NS21	0.999	0	0	0	0	0.001	NS	NS
NS	NS22	1	0	0	0	0	0	NS	NS
NS	NS23	1	0	0	0	0	0	NS	NS
NS	NS24	1	0	0	0	0	0	NS	NS
NS	NS25	1	0	0	0	0	0	NS	NS
NS	NS26	1	0	0	0	0	0	NS	NS
NS	NS27	0.984	0	0	0	0	0.016	NS	NS
NS	NS28	0.994	0	0	0	0	0.006	NS	NS
NS	NS29	0.992	0	0	0	0	0.008	NS	NS
NS	NS30	0.944	0	0	0	0	0.056	NS	NS
NS	NS31	0.773	0	0	0	0	0.227	NS	NS
NS	NS32	0.996	0	0	0	0	0.004	NS	NS
NS	NS33	1	0	0	0	0	0	NS	NS
NS	NS34	0.999	0	0	0	0	0.001	NS	NS
NS	NS35	0.771	0	0	0	0	0.229	NS	NS
NS	NS36	0.981	0	0	0	0	0.019	NS	NS
NS	NS37	0.986	0	0	0	0	0.014	NS	NS
NS	NS38	0.99	0	0	0	0	0.01	NS	NS
NS	NS39	0.99	0	0	0	0	0.01	NS	NS
NS	NS40	1	0	0	0	0	0	NS	NS
DU	DU01	0	1	0	0	0	0	DU	DU
DU	DU02	0	0.822	0	0	0	0.178	DU	DU

Breed	Individual	NS	DU	LR	LW	WB	HY	CACDA	CAT65
DU	DU03	0	1	0	0	0	0	DU	DU
DU	DU04	0	0.862	0	0	0	0.138	DU	DU
DU	DU05	0	1	0	0	0	0	DU	DU
DU	DU06	0	1	0	0	0	0	DU	DU
DU	DU07	0	1	0	0	0	0	DU	DU
DU	DU08	0	1	0	0	0	0	DU	DU
DU	DU09	0	1	0	0	0	0	DU	DU
DU	DU10	0	1	0	0	0	0	DU	DU
DU	DU11	0	1	0	0	0	0	DU	DU
DU	DU12	0	1	0	0	0	0	DU	DU
DU	DU13	0	1	0	0	0	0	DU	DU
DU	DU14	0	0.84	0	0	0	0.16	DU	DU
DU	DU15	0	1	0	0	0	0	DU	DU
DU	DU16	0	1	0	0	0	0	DU	DU
DU	DU17	0	1	0	0	0	0	DU	DU
DU	DU18	0	1	0	0	0	0	DU	DU
DU	DU19	0	1	0	0	0	0	DU	DU
DU	DU20	0	1	0	0	0	0	DU	DU
DU	DU21	0	1	0	0	0	0	DU	DU
DU	DU22	0	1	0	0	0	0	DU	DU
DU	DU23	0	1	0	0	0	0	DU	DU
DU	DU24	0	0.757	0	0	0	0.243	DU	DU
DU	DU25	0	0.934	0	0	0	0.066	DU	DU
DU	DU26	0	1	0	0	0	0	DU	DU
DU	DU27	0	1	0	0	0	0	DU	DU
DU	DU28	0	1	0	0	0	0	DU	DU
DU	DU29	0	1	0	0	0	0	DU	DU
DU	DU30	0	1	0	0	0	0	DU	DU
DU	DU31	0	1	0	0	0	0	DU	DU
DU	DU32	0	1	0	0	0	0	DU	DU
DU	DU33	0	1	0	0	0	0	DU	DU
DU	DU34	0	1	0	0	0	0	DU	DU
DU	DU35	0	1	0	0	0	0	DU	DU
DU	DU36	0	1	0	0	0	0	DU	DU
DU	DU37	0	1	0	0	0	0	DU	DU
DU	DU38	0	1	0	0	0	0	DU	DU
DU	DU39	0	1	0	0	0	0	DU	DU
DU	DU40	0	1	0	0	0	0	DU	DU
LR	LR01	0	0	0.998	0	0	0.002	LR	LR
LR	LR02	0	0	0.999	0	0	0.001	LR	LR
LR	LR03	0	0	0.978	0	0	0.022	LR	LR
LR	LR04	0	0	1	0	0	0	LR	LR
LR	LR05	0	0	0.999	0	0	0.001	LR	LR
LR	LR06	0	0	0.989	0	0	0.011	LR	LR
LR	LR07	0	0	0.999	0	0	0.001	LR	LR

Breed	Individual	NS	DU	LR	LW	WB	HY	CACDA	CAT65
LR	LR08	0	0	0.913	0	0	0.087	LR	LR
LR	LR09	0	0	0.998	0	0	0.002	LR	LR
**LR**	**LR10**	**0**	**0**	**0.568**	**0**	**0**	**0.432**	**LR**	**HY**
LR	LR11	0	0	0.999	0	0	0.001	LR	LR
LR	LR12	0	0	1	0	0	0	LR	LR
LR	LR13	0	0	0.929	0	0	0.071	LR	LR
LR	LR14	0	0	0.868	0	0	0.132	LR	LR
LR	LR15	0	0	0.997	0	0	0.003	LR	LR
LR	LR16	0	0	0.997	0	0	0.003	LR	LR
LR	LR17	0	0	0.964	0	0	0.036	LR	LR
LR	LR18	0	0	0.994	0	0	0.006	LR	LR
LR	LR19	0	0	0.976	0	0	0.024	LR	LR
LR	LR20	0	0	0.999	0	0	0.001	LR	LR
LR	LR21	0	0	0.971	0	0	0.029	LR	LR
LR	LR22	0	0	0.998	0	0	0.002	LR	LR
LR	LR23	0	0	0.997	0	0	0.003	LR	LR
LR	LR24	0	0	0.869	0	0	0.131	LR	LR
LR	LR25	0	0	0.995	0	0	0.005	LR	LR
LR	LR26	0	0	0.986	0	0	0.014	LR	LR
LR	LR27	0	0	0.979	0	0	0.021	LR	LR
LR	LR28	0	0	1	0	0	0	LR	LR
LR	LR29	0	0	0.85	0	0	0.15	LR	LR
LR	LR30	0	0	0.997	0	0	0.003	LR	LR
LR	LR31	0	0	1	0	0	0	LR	LR
LR	LR32	0	0	0.959	0	0	0.041	LR	LR
LR	LR33	0	0	0.716	0	0	0.283	LR	LR
LR	LR34	0	0	0.967	0	0	0.033	LR	LR
LR	LR35	0	0	0.878	0	0	0.122	LR	LR
LR	LR36	0	0	0.988	0	0	0.012	LR	LR
LR	LR37	0	0	0.983	0	0	0.017	LR	LR
LR	LR38	0	0	1	0	0	0	LR	LR
LR	LR39	0	0	0.992	0	0	0.008	LR	LR
LR	LR40	0	0	0.985	0	0	0.015	LR	LR
LW	LW01	0	0	0	1	0	0	LW	LW
**LW**	**LW02**	**0**	**0**	**0**	**0.618**	**0**	**0.382**	**LW**	**HY**
LW	LW03	0	0	0	1	0	0	LW	LW
LW	LW04	0	0	0	0.991	0	0.009	LW	LW
LW	LW05	0	0	0	1	0	0	LW	LW
LW	LW06	0	0	0	0.99	0	0.01	LW	LW
LW	LW07	0	0	0	0.994	0	0.006	LW	LW
LW	LW08	0	0	0	0.737	0	0.263	LW	LW
LW	LW09	0	0	0	0.998	0	0.002	LW	LW
LW	LW10	0	0	0	0.999	0	0.001	LW	LW
LW	LW11	0	0	0	0.999	0	0.001	LW	LW
LW	LW12	0	0	0	1	0	0	LW	LW

Breed	Individual	NS	DU	LR	LW	WB	HY	CACDA	CAT65
LW	LW13	0	0	0	0.989	0	0.011	LW	LW
LW	LW14	0	0	0	0.998	0	0.002	LW	LW
LW	LW15	0	0	0	1	0	0	LW	LW
LW	LW16	0	0	0	0.999	0	0.001	LW	LW
LW	LW17	0	0	0	1	0	0	LW	LW
LW	LW18	0	0	0	0.999	0	0.001	LW	LW
LW	LW19	0	0	0	0.994	0	0.006	LW	LW
LW	LW20	0	0	0	0.999	0	0.001	LW	LW
LW	LW21	0	0	0	0.988	0	0.012	LW	LW
LW	LW22	0	0	0	0.999	0	0.001	LW	LW
LW	LW23	0	0	0	0.998	0	0.002	LW	LW
LW	LW24	0	0	0	0.998	0	0.002	LW	LW
LW	LW25	0	0	0	0.999	0	0.001	LW	LW
LW	LW26	0	0	0	0.991	0	0.009	LW	LW
LW	LW27	0	0	0	0.994	0	0.006	LW	LW
LW	LW28	0	0	0	1	0	0	LW	LW
LW	LW29	0	0	0	0.998	0	0.002	LW	LW
LW	LW30	0	0	0	0.972	0	0.028	LW	LW
LW	LW31	0	0	0	0.997	0	0.003	LW	LW
LW	LW32	0	0	0	0.996	0	0.004	LW	LW
LW	LW33	0	0	0	1	0	0	LW	LW
LW	LW34	0	0	0	0.992	0	0.008	LW	LW
LW	LW35	0	0	0	0.985	0	0.015	LW	LW
LW	LW36	0	0	0	0.999	0	0.001	LW	LW
LW	LW37	0	0	0	0.999	0	0.001	LW	LW
LW	LW38	0	0	0	0.996	0	0.004	LW	LW
LW	LW39	0	0	0	1	0	0	LW	LW
LW	LW40	0	0	0	0.966	0	0.034	LW	LW
WB	WB01	0	0	0	0	0.999	0.001	WB	WB
WB	WB02	0	0	0	0	1	0	WB	WB
WB	WB03	0	0	0	0	0.997	0.003	WB	WB
WB	WB04	0	0	0	0	1	0	WB	WB
WB	WB05	0	0	0	0	0.999	0.001	WB	WB
WB	WB06	0	0	0	0	1	0	WB	WB
WB	WB07	0	0	0	0	0.999	0.001	WB	WB
WB	WB08	0	0	0	0	0.999	0.001	WB	WB
WB	WB09	0	0	0	0	1	0	WB	WB
WB	WB10	0	0	0	0	0.999	0.001	WB	WB
WB	WB11	0	0	0	0	1	0	WB	WB
WB	WB12	0	0	0	0	1	0	WB	WB
WB	WB13	0	0	0	0	1	0	WB	WB
WB	WB14	0	0	0	0	0.999	0.001	WB	WB
WB	WB15	0	0	0	0	0.999	0.001	WB	WB
WB	WB16	0	0	0	0	0.997	0.003	WB	WB
WB	WB17	0	0	0	0	1	0	WB	WB

Breed	Individual	NS	DU	LR	LW	WB	HY	CACDA	CAT65
WB	WB18	0	0	0	0	1	0	WB	WB
WB	WB19	0	0	0	0	0.998	0.002	WB	WB
WB	WB20	0	0	0	0	0.999	0.001	WB	WB
WB	WB21	0	0	0	0	0.999	0.001	WB	WB
WB	WB22	0	0	0	0	1	0	WB	WB
WB	WB23	0	0	0	0	0.999	0.001	WB	WB
WB	WB24	0	0	0	0	0.999	0.001	WB	WB
WB	WB25	0	0	0	0	0.999	0.001	WB	WB
WB	WB26	0	0	0	0	1	0	WB	WB
WB	WB27	0	0	0	0	1	0	WB	WB
WB	WB28	0	0	0	0	0.982	0.018	WB	WB
WB	WB29	0	0	0	0	1	0	WB	WB
WB	WB30	0	0	0	0	1	0	WB	WB
WB	WB31	0	0	0	0	0.999	0.001	WB	WB
WB	WB32	0	0	0	0	1	0	WB	WB
WB	WB33	0	0	0	0	0.999	0.001	WB	WB
WB	WB34	0	0	0	0	1	0	WB	WB
WB	WB35	0	0	0	0	0.999	0.001	WB	WB
WB	WB36	0	0	0	0	0.998	0.002	WB	WB
WB	WB37	0	0	0	0	0.999	0.001	WB	WB
WB	WB38	0	0	0	0	1	0	WB	WB
WB	WB39	0	0	0	0	1	0	WB	WB
WB	WB40	0	0	0	0	0.999	0.001	WB	WB
NSxDU	NSxDU21	0.009	0.1	0	0	0	0.892	HY	HY
NSxDU	NSxDU22	0.001	0.038	0	0	0	0.961	HY	HY
NSxDU	NSxDU23	0.082	0.088	0	0	0	0.83	HY	HY
NSxDU	NSxDU24	0.344	0	0	0	0	0.656	HY	HY
**NSxDU**	**NSxDU25**	**0.004**	**0.606**	**0**	**0**	**0**	**0.39**	**DU**	**HY**
NSxDU	NSxDU26	0.036	0.046	0	0	0	0.918	HY	HY
NSxDU	NSxDU27	0.001	0.014	0	0	0	0.986	HY	HY
NSxDU	NSxDU28	0.006	0.103	0	0	0	0.891	HY	HY
NSxDU	NSxDU29	0.004	0.002	0.001	0	0	0.993	HY	HY
NSxDU	NSxDU30	0.072	0.019	0	0	0	0.909	HY	HY
NSxLR	NSxLR21	0.025	0	0.357	0	0	0.617	HY	HY
NSxLR	NSxLR22	0.002	0	0.031	0	0	0.966	HY	HY
NSxLR	NSxLR23	0.135	0	0.219	0	0	0.646	HY	HY
NSxLR	NSxLR24	0.049	0	0.001	0	0	0.951	HY	HY
NSxLR	NSxLR25	0.007	0	0.211	0	0	0.782	HY	HY
**NSxLR**	**NSxLR26**	**0.584**	**0**	**0.113**	**0**	**0**	**0.303**	**NS**	**HY**
NSxLR	NSxLR27	0.004	0	0.016	0	0	0.98	HY	HY
NSxLR	NSxLR28	0.009	0	0.274	0	0	0.718	HY	HY
NSxLR	NSxLR29	0.002	0	0.089	0	0.001	0.908	HY	HY
NSxLR	NSxLR30	0.004	0	0.005	0	0	0.991	HY	HY
NSxLW	NSxLW21	0.2	0	0.001	0.002	0	0.798	HY	HY
NSxLW	NSxLW22	0.342	0	0	0.028	0	0.63	HY	HY

Breed	Individual	NS	DU	LR	LW	WB	HY	CACDA	CAT65
NSxLW	NSxLW23	0.007	0	0	0.003	0	0.99	HY	HY
NSxLW	NSxLW24	0.002	0	0	0.201	0	0.797	HY	HY
NSxLW	NSxLW25	0.261	0	0	0.153	0	0.586	HY	HY
NSxLW	NSxLW26	0.018	0	0	0.416	0	0.566	HY	HY
NSxLW	NSxLW27	0.018	0	0	0.078	0	0.904	HY	HY
NSxLW	NSxLW28	0.195	0	0	0	0	0.805	HY	HY
NSxLW	NSxLW29	0.118	0	0.004	0.017	0	0.861	HY	HY
NSxLW	NSxLW30	0.06	0	0	0.129	0	0.811	HY	HY
**NSxWB**	**NSxWB21**	**0.457**	**0**	**0**	**0**	**0.116**	**0.427**	**NS**	**HY**
**NSxWB**	**NSxWB22**	**0.008**	**0**	**0**	**0**	**0.627**	**0.366**	**WB**	**HY**
NSxWB	NSxWB23	0.002	0	0.002	0.001	0	0.995	HY	HY
NSxWB	NSxWB24	0.033	0	0	0	0.046	0.921	HY	HY
**NSxWB**	**NSxWB25**	**0**	**0**	**0**	**0**	**0.503**	**0.497**	**HY**	**HY**
NSxWB	NSxWB26	0.305	0	0	0	0.001	0.694	HY	HY
NSxWB	NSxWB27	0.01	0	0	0	0.194	0.796	HY	HY
NSxWB	NSxWB28	0.006	0	0	0	0.029	0.965	HY	HY
NSxWB	NSxWB29	0.472	0	0	0	0.026	0.502	HY	HY
**NSxWB**	**NSxWB30**	**0.519**	**0**	**0**	**0**	**0.12**	**0.36**	**NS**	**HY**
DUxLR	DUxLR21	0	0.038	0	0	0	0.961	HY	HY
DUxLR	DUxLR22	0	0.005	0.003	0	0	0.992	HY	HY
DUxLR	DUxLR23	0	0.003	0.01	0	0	0.987	HY	HY
DUxLR	DUxLR24	0	0.05	0.037	0	0	0.913	HY	HY
DUxLR	DUxLR25	0	0.006	0.163	0	0	0.831	HY	HY
DUxLR	DUxLR26	0	0.014	0.05	0	0	0.936	HY	HY
DUxLR	DUxLR27	0	0.006	0.006	0	0	0.989	HY	HY
DUxLR	DUxLR28	0	0.027	0.026	0	0	0.947	HY	HY
DUxLR	DUxLR29	0	0.014	0.015	0	0	0.971	HY	HY
DUxLR	DUxLR30	0	0.022	0.024	0	0	0.954	HY	HY
DUxLW	DUxLW21	0	0.052	0	0.001	0.001	0.947	HY	HY
DUxLW	DUxLW22	0	0.012	0	0.361	0	0.626	HY	HY
DUxLW	DUxLW23	0	0.018	0	0.002	0	0.98	HY	HY
DUxLW	DUxLW24	0	0.029	0	0.023	0	0.948	HY	HY
DUxLW	DUxLW25	0	0.029	0	0.056	0	0.914	HY	HY
DUxLW	DUxLW26	0	0.466	0	0	0	0.534	HY	HY
DUxLW	DUxLW27	0	0.053	0	0.088	0	0.859	HY	HY
DUxLW	DUxLW28	0	0	0	0.103	0	0.897	HY	HY
DUxLW	DUxLW29	0	0.02	0	0.398	0	0.582	HY	HY
DUxLW	DUxLW30	0.002	0.017	0	0	0	0.98	HY	HY
DUxWB	DUxWB21	0	0.101	0	0	0.002	0.896	HY	HY
DUxWB	DUxWB22	0	0.093	0	0	0.029	0.877	HY	HY
DUxWB	DUxWB23	0	0.032	0	0	0.004	0.964	HY	HY
DUxWB	DUxWB24	0	0.03	0	0	0.003	0.967	HY	HY
DUxWB	DUxWB25	0	0.013	0	0	0.031	0.956	HY	HY
DUxWB	DUxWB26	0	0.035	0	0	0.041	0.924	HY	HY
DUxWB	DUxWB27	0	0.116	0	0	0.019	0.865	HY	HY

Breed	Individual	NS	DU	LR	LW	WB	HY	CACDA	CAT65
DUxWB	DUxWB28	0	0.086	0	0	0.002	0.912	HY	HY
DUxWB	DUxWB29	0	0.029	0	0	0.015	0.956	HY	HY
DUxWB	DUxWB30	0	0.063	0	0	0.012	0.924	HY	HY
**LRxLW**	**LRxLW21**	**0**	**0**	**0.022**	**0.924**	**0**	**0.054**	**LW**	**LW**
**LRxLW**	**LRxLW22**	**0**	**0**	**0.05**	**0.826**	**0**	**0.124**	**LW**	**LW**
LRxLW	LRxLW23	0	0	0.027	0.022	0	0.95	HY	HY
LRxLW	LRxLW24	0	0	0.856	0	0	0.144	**LR**	**LR**
**LRxLW**	**LRxLW25**	**0**	**0**	**0.59**	**0.064**	**0**	**0.346**	**LR**	**HY**
LRxLW	LRxLW26	0	0	0.005	0.034	0	0.961	HY	HY
**LRxLW**	**LRxLW27**	**0**	**0**	**0.567**	**0.007**	**0**	**0.425**	**LR**	**HY**
**LRxLW**	**LRxLW28**	**0**	**0**	**0.067**	**0.581**	**0**	**0.352**	**LW**	**HY**
LRxLW	LRxLW29	0	0	0.257	0	0	0.742	HY	HY
LRxLW	LRxLW30	0	0	0.011	0.159	0	0.831	HY	HY
LRxWB	LRxWB21	0	0	0.12	0	0.151	0.729	HY	HY
LRxWB	LRxWB22	0	0	0.184	0	0.002	0.813	HY	HY
**LRxWB**	**LRxWB23**	**0**	**0**	**0.013**	**0**	**0.576**	**0.411**	**WB**	**HY**
**LRxWB**	**LRxWB24**	**0**	**0**	**0.127**	**0**	**0.777**	**0.096**	**WB**	**WB**
LRxWB	LRxWB25	0	0	0.213	0	0.212	0.575	HY	HY
**LRxWB**	**LRxWB26**	**0**	**0**	**0.783**	**0**	**0.01**	**0.208**	**LR**	**LR**
LRxWB	LRxWB27	0	0	0.069	0	0.002	0.929	HY	HY
LRxWB	LRxWB28	0	0	0.011	0	0.085	0.904	HY	HY
LRxWB	LRxWB29	0	0	0.404	0	0.03	0.567	HY	HY
LRxWB	LRxWB30	0	0	0.229	0	0.004	0.767	HY	HY
**LWxWB**	**LWxWB21**	**0**	**0**	**0**	**0**	**0.634**	**0.366**	**WB**	**HY**
LWxWB	LWxWB22	0	0	0.001	0.001	0.226	0.772	HY	HY
LWxWB	LWxWB23	0	0	0	0.096	0.021	0.883	HY	HY
LWxWB	LWxWB24	0	0	0	0.032	0.046	0.922	HY	HY
LWxWB	LWxWB25	0	0	0	0.001	0.086	0.913	HY	HY
LWxWB	LWxWB26	0	0	0	0.002	0.044	0.954	HY	HY
LWxWB	LWxWB27	0	0	0	0.004	0.025	0.971	HY	HY
LWxWB	LWxWB28	0	0	0	0.001	0.043	0.957	HY	HY
LWxWB	LWxWB29	0	0	0	0.413	0.02	0.567	HY	HY
LWxWB	LWxWB30	0	0	0	0.01	0.118	0.873	HY	HY
Mean Assignation probability		0.981	0.980	0.957	0.979	0.999	0.767		
				0.979			0.767		
				0.908					

[1]Breeds: NS = Nero Siciliano; DU = Duroc; LW = Large White; LR = Landrace; WB = Wild Boar; the hybrids of each breeds' couple are identified by the two breeds separated by "x".
